# Summer Sessions

**Published:** 1875-08

**Authors:** 


### EDITORIAL, ETC.

---

*Summer Sessions.*—For a number of years past a spring and summer course of instruction has been carried on at the Baltimore College of Dental Surgery. Until the present course the students in attendance have been under the charge of a Demonstrator, and the instruction confined to practice only.

The present session, however, is held under the supervision of Prof. James B. Hodgkin, Professor of Dental Mechanism and Metallurgy, and is attended by quite a number of matriculants who purpose attending also the regular sessions of the College. The benefits of such a preliminary course of instruction cannot be over-estimated, and its results will be of inestimable value to students, who after availing themselves of such an opportunity will be the better enabled to commence the winter course of study and practice.

The attention of those who desire to commence the study of a profession, without any previous experience, or at least possessing only what may be obtained in a private office, which at best is in most cases limited, is called to the many advantages to be derived from such a spring and summer course of preliminary instruction.

No private office can present the same facilities for practice, etc., to the dental student, and such a course of study and practice will supply a want long felt by many in their endeavors to perfect themselves in their chosen profession.

